# In Situ Contact-Separation TENG for High-Speed Rail Wind Monitoring

**DOI:** 10.3390/nano15110839

**Published:** 2025-05-30

**Authors:** Guangzheng Wang, Depeng Fu, Yuankun Li, Xiaoxiong Wang

**Affiliations:** 1 College of Physics, Qingdao University, Qingdao 266071, China; wgz_alpha@163.com (G.W.); fdpdffzy@163.com (D.F.); liyuankun328@163.com (Y.L.); 2 University-Industry Joint Center for Ocean Observation and Broadband Communication, College of Physics, Qingdao University, Qingdao 266071, China; 3 Weihai Innovation Research Institute of Qingdao University, Weihai 264200, China

**Keywords:** nanogenerators, self-powered, electrospinning, wind speed monitoring

## Abstract

Triboelectric nanogenerators have attracted extensive attention as they can complete sensing during energy conversion, triggering a series of self-powered designs. Traditional TENG bipolar independent fabrication technology requires secondary motion control, which limits its application scenarios. In this work, we propose a flag-type TENG prepared using in situ electrospinning technology, in which the connecting region is obtained by electrospinning deposition of PVDF on nylon as the receiving electrode. The active area is isolated with silicone oil paper. After electrospinning, the silicone oil paper was removed, and the distance between the nylon and PVDF is far beyond the van der Waals range. Thus, contact separation can be effectively carried out under the action of wind. The device has been proven to be able to be used for monitoring wind conditions at high-speed rail stations and enables completely self-powered monitoring of the wind level using self-powered LED coding. The device no longer relies on additional batteries or wires to work, providing additional ideas for future self-powered system design.

## 1. Introduction

With the rapid development of the Internet of Things and smart sensors, there is an urgent need for the design of self-powered systems. In 2006, Wang Zhonglin’s team first developed nanogenerator technology based on the piezoelectric effect [1]. In 2012, they successfully developed a new type of nanogenerator, namely, the triboelectric nanogenerator (TENG), by introducing the triboelectric effect, and its energy conversion efficiency was several times higher than that of early piezoelectric products [2]. This breakthrough not only promotes the innovation of nano-energy technology, but also gives birth to the emerging research field of self-powered sensor systems, which provide key technical support for the low-power operation of IoT terminal equipment. Although these works have initially formed the prototype of the self-powered system, achieving effective energy consumption matching during the application process is still a key scientific challenge for realizing system operation independent of the traditional energy system. Taking low-power STM32 as an example, its power consumption is about 10 mW. The power consumption of the Bluetooth transceiver chip is also on the order of 10 mW. At present, the typical output power density of the nanogenerator is on the order of 10 W/m^2^. Considering the actual loss, its equivalent DC output power is about 1 W/m^2^. Therefore, for a 10 mW chip, the area required for a single chip drive has reached the order of 100 cm^2^. The current mainstream research direction is located in the wearable field, and sufficient energy for the support system is obtained through large-area fabric wear and rich human energy collection. In fact, using a reasonable application scenario design, a fully self-powered system that does not rely on the second TENG can be designed [3,4,5,6,7,8,9,10,11,12,13].

Electrospinning technology has long been used in the design process of nanogenerators. Electrospinning technology is known for its rich microstructure design methods, and in situ electrospinning technology has attracted wide attention for its convenience [4]. Here, in situ means that the fiber is not deposited on the electrode and then peeled off after the deposition process. Rather, it is directly deposited on the surface of the load medium by using the space electric field induction. At this time, even if the medium is an insulator, the related electrospinning process can be completed. In this way, a good adhesion effect is obtained without further assembly. Therefore, in situ electrospinning has many applications in the design and preparation of wound dressings [14,15,16,17,18,19,20].

The development of high-speed railways has a huge driving effect on China’s economy [21,22,23,24,25]. However, the increase in high-speed railway speed has introduced a series of engineering requirements. In the process of a high-speed train passing through the station, if the train does not stop, it will produce a large wind pressure, which will affect the roof structure of the high-speed railway station. Therefore, the wind pressure of the train needs to be monitored, especially when the natural wind speed is superimposed. The wind pressure needs real-time monitoring to adjust the speed of high-speed railway passing through the station [26,27,28]. However, high-speed rail uses tens of thousands of volts and thousands of amperes of current electrical systems [29,30,31,32]. Therefore, any wiring, even weak current lines, will introduce the risk of short circuits caused by high-voltage breakdown and creepage, which seriously endangers the operation of high-speed rail lines. Therefore, the railway department has proposed the design requirements of a fully self-powered sensing system.

Traditional electrospinning typically involves first collecting nanofibers on a universal substrate such as metal foil or silicone oil paper, followed by integration into target devices through steps like detachment, transfer, and bonding. However, this process may lead to structural damage to the fiber membrane and insufficient interfacial adhesion. In contrast, in situ electrospinning technology enables direct deposition of fibers in specific regions of the target device. For example, in the flexible structural parts of the TENG that do not undergo contact separation, in situ electrospinning eliminates the need for transfer and bonding steps, improving interfacial adhesion and device structural stability. Fiber membranes prepared with traditional electrospinning are bonded to device substrates via physical fitting or adhesives, resulting in weak interfacial adhesion that may cause delamination or failure due to friction or bending during long-term use.

In this work, we obtained a flag-type TENG using in situ electrospinning technology. By changing the flag area to generate a response to different wind pressures, we used the contact separation caused by a specific wind pressure to light up the LED, and we achieved a sensing that does not depend on any battery and wiring. The wind sensing results can be observed by the human eye and also can be read by optical communication terminals such as CCD. The design of the related self-powered sensing system greatly ensures the safety of railway operation.

## 2. Materials and Methods

Polyvinylidene fluoride (PVDF Mw ≈ 100 w) was purchased from Shanghai Sanai Fuxin Material Technology Co., Ltd. (Shanghai, China). Nylon 6 (PA6) was purchased from McLean Biochemical Technology Co., Ltd. (Shanghai, China). Analytically pure acetone, N, N-dimethylformamide (DMF), and hexafluoroisopropanol (HFIP) was purchased from Shanghai Macklin Biochemical Co., Ltd. (Shanghai, China).

**Figure 1:**
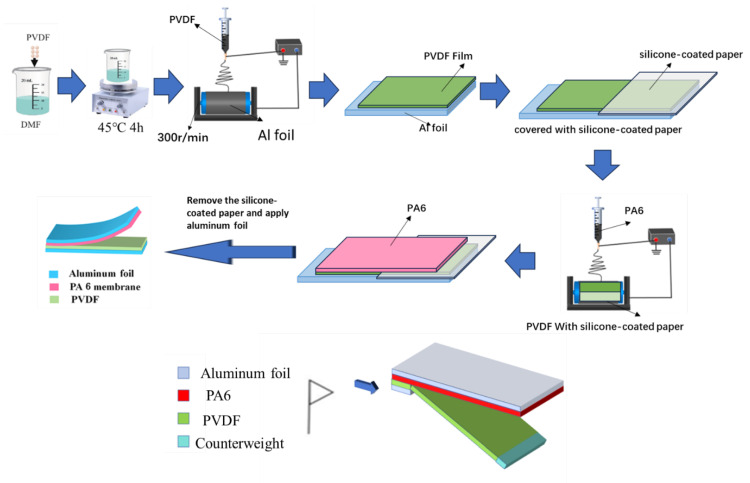
Flow chart of the preparation of the in situ fiber membrane structure.

To prepare the polyvinylidene fluoride (PVDF) solution, PVDF powder was dissolved in a mixed solvent of acetone and N,N-dimethylformamide (DMF) (mass ratio 1:1) under continuous stirring at 45 °C for 4 h, yielding a homogeneous solution with a concentration of 10 wt%. For the nylon 6 (PA6) solution, PA6 powder was dissolved in hexafluoroisopropanol (HFIP) solvent and stirred at room temperature for 4 h to obtain an 8 wt% uniform solution. The preparation of nanofiber membranes is illustrated in Figure 1. Polyvinylidene fluoride (PVDF) thin films were successfully fabricated on aluminum foil using the electrospinning method. During the process, the ambient temperature was maintained at 25 ± 3 °C, and the atmospheric humidity was stabilized within a range of 42 ± 5%. The feed rate of the polymer solution was set to 1 mL/h, and the electrospinning voltage was adjusted to 13 kV. The distance between the syringe needle tip and the drum collector was precisely controlled at 15 cm, while the rotational speed of the drum collector was set to 300 r/min. To fabricate a composite membrane of polyvinylidene fluoride (PVDF) and nylon 6 (PA6), a layer of nylon 6 (PA6) film was in situ formed on the prepared PVDF film. To achieve the contact-separation effect at the other end, half of the PVDF film was covered with silicone paper. Thus, the PA6 film was in situ formed on half of the PVDF film and on half of the silicone paper. After the electrospinning process was completed, the silicone paper was removed, and an in situ-formed triboelectric nanogenerator (TENG) was obtained.

During the electrospinning of PA6 fibers, the temperature was maintained at around 15 °C with a fluctuation range of no more than ± 3 °C. The collecting plate should be placed at a fixed position 15 cm away from the needle. The rotational speed of the drum collector is 300 r/min.

Meanwhile, the atmospheric humidity was controlled at approximately 35% with an allowable fluctuation of ±5%. The flow rate during the electrospinning process was set at 6 mL/h, and the electrospinning voltage was kept at 16 kV, as shown in Figure 1.

Scanning electron microscopy (SEM, Zeiss Sigma 500/VP model) was used to observe the surface morphology and microstructure of the TENG. Fourier transform infrared (FTIR) spectroscopy (VERTEX80v) and Raman spectroscopy (Nicolet 5700) were employed for qualitative identification, quantitative analysis, and structural characterization of the TENG components. Additionally, ImageJ software was used to measure the diameters of the electrospun fiber membranes to determine the average fiber diameter. The surface morphology of the nanofibers was also examined. The thickness of the thin films was measured using a digital micrometer thickness gauge (Koslo). A programmable electrometer (Keithley 6514) was utilized to characterize the electrical output performance, including open-circuit voltage, short-circuit current, and short-circuit charge transfer.

## 3. Results and Discussion

**Figure 2:**
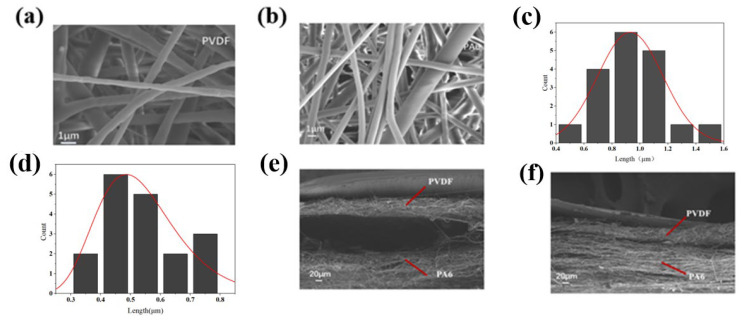
Characterization of the TENG. (**a**) SEM imaging was performed on PVDF. (**b**) SEM imaging was performed on PA6. (**c**) Fiber diameter distribution of PA6. (**d**) Fiber diameter distribution of PVDF. (**e**) Cross-sectional SEM images of the in situ region of the TENG. (**f**) Cross-sectional SEM images of the ex situ region of the TENG.

**Sample characterization:** The surface SEM images of PVDF and PA6 nanofiber membranes with their fiber morphology are illustrated in Figure 2a,b. The SEM images were analyzed, and the fiber diameters of PVDF and PA6 were statistically analyzed using Image J software. The PVDF fiber diameter is concentrated in the range of 0.4–0.5 μm, while the PA6 fiber diameter is concentrated in the range of 0.8–1.0 μm, showing significant differences in diameter characteristics. The thickness of the PVDF film in the ex situ region was measured with a digital micrometer thickness gauge (Koslo). The PVDF film thickness is 0.077 mm, the PA6 film thickness is 0.368 mm, and the thickness of the adhesive region of PVDF and PA6 in the in situ region is 0.044 mm. The cross-sectional SEM images of PVDF and PA6 membranes formed in situ and ex situ reveal a certain degree of fiber entanglement between the two layers of the in situ fiber membranes. It can be observed that the two-layer fibrous PVDF and PA6 membranes exhibit close interfacial contact with partial fiber interpenetration between the layers. The indistinct boundaries between the fibers, along with the observed interpenetration and entanglement, suggest pronounced interfacial interactions between the two fibrous layers during the in situ fabrication process. The ex situ two-layer fibrous membranes, however, exhibit a distinct bilayer separation without interfacial fiber integration. As shown in Figure 2e,f, the in situ fabrication process initiates interfacial interactions between the two fibrous membranes during their formation, potentially leading to partial fusion at the interface, which may positively enhance interfacial adhesion between the layers. In contrast, the ex situ fabricated membranes exhibit independent interfacial layers, while the ex situ regions are structurally compatible with the contact-separation requirements of TENG applications.

**Figure 3:**
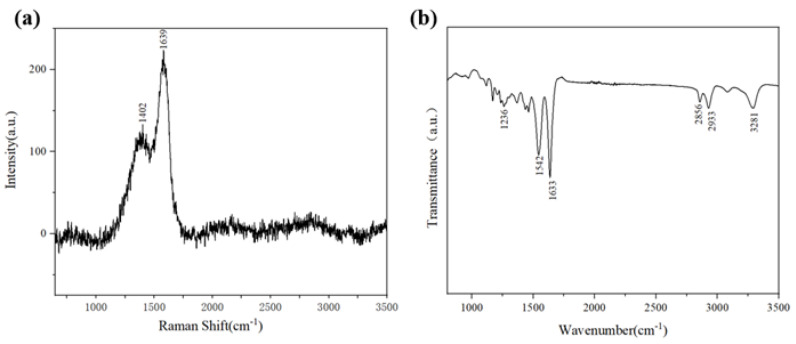
(**a**) Raman spectra of PA6. (**b**) FTIR spectrum of PA6.

Figure 3a presents the Raman spectrum of PA6, where characteristic peaks in the 2900–3000 cm^−1^ range are attributed to the symmetric and asymmetric stretching vibrations of C–H bonds in methylene chains (–(CH_2_)_5_–), indicating the regularity of aliphatic chain structures. The peak near 1640 cm^−1^ corresponds to the C=O stretching vibration of amide groups (–CONH–, amide I band), while the intense peak at 1440 cm^−1^ originates from CH_2_ scissoring vibrations (δ(CH_2_)) or C–C backbone vibrations, reflecting the conformational features of the molecular main chain.

In the FTIR spectrum shown in Figure 3b, the strong absorption band near 3300 cm^−1^ is assigned to the N–H stretching vibration of amide groups, with broadening likely associated with hydrogen bonding interactions. The peaks at 1640 cm^−1^ and 1540 cm^−1^ correspond to the amide I band (C=O stretching) and amide II band (coupled N–H in-plane bending and C–N stretching), respectively. Absorptions below 1450 cm^−1^ primarily stem from C–H bending vibrations of methylene chains and C–C backbone vibrations. The synergistic analysis of Raman and FTIR spectra confirms that the molecular structure of PA6 consists of polar amide groups and nonpolar methylene chains.

**Electrical Performance of the TENG:** The selection of these two nanofiber membranes (PVDF and PA6) is based on the difference in their electron affinities. In the initial state of the TENG, the PVDF and PA6 membranes are in close contact. Due to their distinct surface electron affinities, electrons migrate from the PA6 surface to the PVDF surface, resulting in the PA6 nanofiber membrane surface becoming positively charged and the PVDF nanofiber membrane surface becoming negatively charged. However, at this stage, although charge separation has occurred, no current or potential difference exists in the system. When the positive and negative tribo-layers begin to separate, this charge separation induces a potential difference across the conductive fabric. This potential difference provides the driving force for electrons to flow from the lower electrode to the upper electrode. As the two charged surfaces further separate, the potential difference gradually increases until reaching a maximum value. At this point, short-circuiting the electrodes allows electrons to flow driven by the potential difference, thereby generating an electric current. When the positive and negative tribo-layers reach the maximum separation distance and equilibrium is established, the open-circuit voltage drops to zero. This occurs as electron migration stabilizes and charge separation reaches a dynamic equilibrium. Upon re-approaching the two charged surfaces, a reverse current is generated in the system. During the re-contact process, electrons begin to migrate from the PVDF surface back to the PA6 surface, altering the charge separation state. The resulting current flows in the opposite direction to the previous one, forming a reverse current.

**Figure 4:**
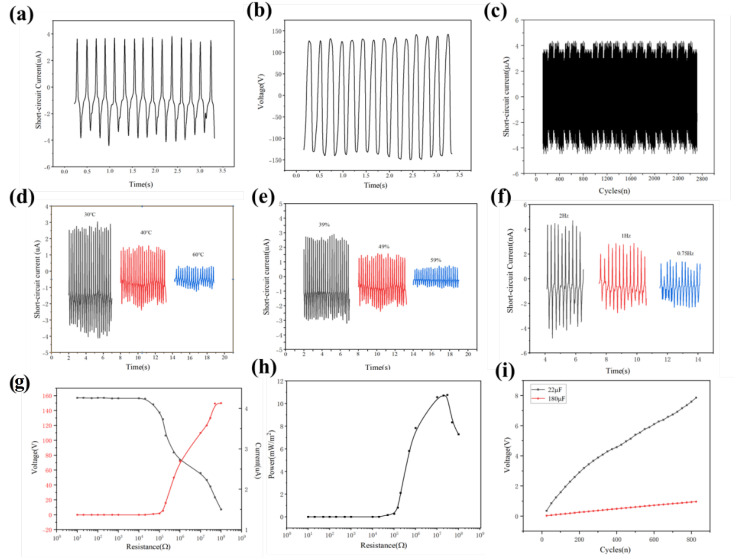
Electrical properties of the TENG. (**a**) Short-circuit current. (**b**) Open-circuit voltage. (**c**) Stability and durability test of the TENG. The environmental adaptability of the TENG was tested at (**d**) different environmental temperatures. (**e**) Different environmental humidity levels and (**f**) short-circuit current of different impact frequencies. (**g**) The TENG outputs current and voltage under different external load resistance conditions. (**h**) The relationship between output power and load resistance. (**i**) Charging curve of the TENG to commercial capacitor.

To characterize the electrical output performance of the fabricated TENG, a device with an active area of 64 cm^2^ was fabricated, and a series of electrical signals were measured. As shown in Figure 4a,b, the TENG achieved a short-circuit current (Isc) of 4 μA and an open-circuit voltage (Voc) of 150 V, demonstrating superior output performance. For practical applications, the long-term stability and reliability of the triboelectric nanogenerator were evaluated with 2800-cycle testing, as shown in Figure 4c. After repeated cycles, no significant degradation in short-circuit current was observed, confirming the excellent durability of the TENG. Of course, environmental factors are also critical. The TENG was tested under different temperatures, humidity levels, and impact frequencies.

In the complex operational environment of high-speed railways, environmental factors play a crucial role in influencing the performance of triboelectric nanogenerators (TENGs). Performance tests were conducted on the TENG under typical scenarios such as temperature fluctuations, humidity changes, and differences in wind impact frequency on the roof caused by high-speed trains passing through stations, which are common in high-speed rail operations. When trains traverse different regional climate zones, changes in ambient temperature significantly affect TENG performance. Measured data show that as the ambient temperature rises (e.g., from low winter temperatures in North China to high summer temperatures in South China), the short-circuit current of the TENG exhibits a gradual decreasing trend (as shown in Figure 4d). This characteristic requires special attention in scenarios such as power supply for high-speed rail air conditioning systems and large temperature gradients inside/outside carriages to ensure stable power output across temperature variations. Changes in humidity also impact TENG performance. Under typical high-humidity environments such as the southern plum rain season, the short-circuit current of the TENG gradually decreases with increasing ambient humidity (as shown in Figure 4e). In the aerodynamic environment of high-speed rail operations, periodic wind impacts generated by trains passing through stations at high speed become a key factor affecting TENG performance. The variation in wind impact frequency directly influences the TENG devices installed on station roofs. Specifically, the short-circuit current of TENGs increases proportionally with changes in impact frequency; under the same charge transfer conditions, higher impact frequencies (e.g., during high-speed passage through stations) shorten the duration of current peaks, thereby increasing the amplitude of short-circuit current (as shown in Figure 4f). This characteristic is closely related to wind speeds generated by high-speed trains at different operating speeds, providing critical reference value for the frequency adaptability design of vehicle-mounted energy recovery systems.

To investigate the TENG’s output capability under varying loads, researchers characterized the relationship between electrical signals and load resistance. As depicted in Figure 4g, the generated voltage of the triboelectric nanogenerator increases steadily with rising load resistance, whereas the output current exhibits an inverse trend—decreasing as the resistance increases. To further evaluate the TENG’s performance, the output power under different loads was calculated using the formula P = UI, and the specific power density (power per unit area) was obtained by normalizing the calculated power with the device’s effective contact area. As shown in Figure 4h, the maximum specific power density reached 11.14 mW/m^2^ at a load resistance of 10^7^ Ω. Additionally, to demonstrate the practical application potential of the TENG, its pulsed energy was stored in a capacitor. As shown in Figure 4i, a commercial 22 μF capacitor was charged to 8 V; this voltage is sufficient to continuously power many small portable smart electronic devices, such as sensors and LED lights. Therefore, the TENG exhibits significant development advantages in the field of portable self-powered power sources. In summary, the comprehensive evaluation of the TENG’s electrical performance confirms that it can serve as a self-powered power source, efficiently converting mechanical energy into electrical energy.

**Figure 5:**
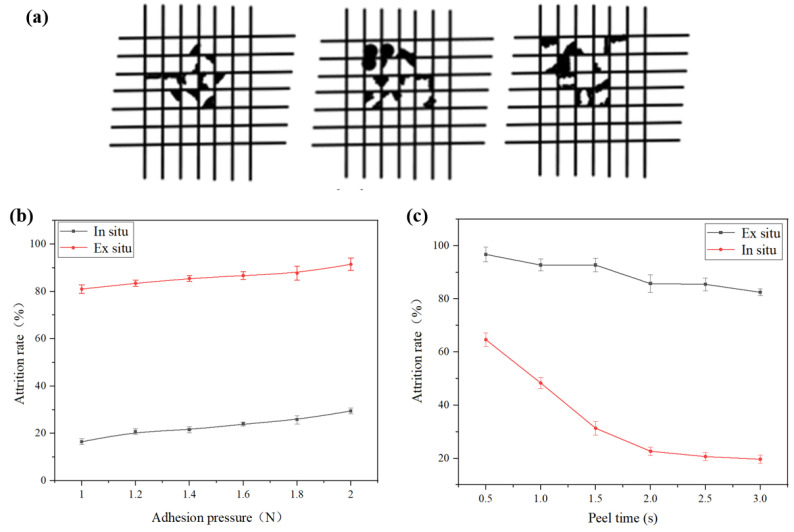
(**a**) Schematic diagram of the several grids method. (**b**) Attrition rates of in situ and ex situ films under different adhesive pressures. (**c**) Attrition rates of in situ and ex situ films under different peeling times.

**Several grids method for measuring adhesion of in situ film:** The several grids method (cross-cut test) is a widely used technique for evaluating the adhesion of thin films. This method involves creating a standardized grid pattern on the film surface using precision cutting, followed by adhesive tape application and rapid peeling to assess the detachment of film fragments from the substrate. The procedure provides a visual quantification of interfacial adhesion performance. In experimental implementation, a several-grids blade was vertically aligned to the film surface to ensure full-thickness incisions (penetrating the film without excessive substrate damage). Post-cutting, residual particulates were removed using soft bristle brushes and compressed air to eliminate interfacial contamination. A pressure-sensitive tape was then uniformly applied to completely cover the grid area, ensuring optimal contact through controlled pressure. To investigate adhesion differences between in situ and ex situ film regions, we systematically varied two parameters: peeling duration (0.5–5.0 s) under constant adhesion pressure (1.5 N/cm^2^) and adhesion pressure (0.5–3.0 N/cm^2^) at fixed peeling duration (2.0 s). As shown in Figure 5a, in situ regions demonstrated significantly lower detachment rates compared to ex situ areas (*p* < 0.05) under equivalent pressure conditions, with all test regions exhibiting time-dependent detachment reduction until stabilization at 20% after approximately 2.5 s. Pressure-dependent measurements (Figure 5b) revealed a positive correlation between adhesion pressure and detachment rates across all samples, while maintaining the superior performance of in situ deposited films throughout the pressure gradient (Δrate = 12.3 ± 1.8% vs. 34.7 ± 2.5% at 3.0 N/cm^2^). This methodology effectively discriminates interfacial adhesion characteristics through controlled mechanical stimuli, demonstrating the enhanced bonding integrity of in situ fabricated films compared to ex situ counterparts. The several grids method is a standardized method for evaluating the adhesion of thin film materials, as shown in Figure 5a, ensuring their reliability and durability. To assess the adhesion of in situ PVDF and PA6 films, three sets of experiments using the several grids method were conducted. Here, a 10 × 10 grid was scribed on the film surface, followed by tape peeling tests. When varying adhesive pressure while keeping the peeling time constant at 2 s, the in situ region again exhibited a lower detachment rate than the ex situ region, with detachment rates increasing with higher adhesive pressure, as demonstrated in Figure 5b. Under identical adhesive pressure, the effect of peeling time was investigated. As shown in Figure 5c, the detachment rate of the in situ region was significantly lower than that of the ex situ region. Across all regions, the detachment rate decreased gradually with increasing peeling time, stabilizing at 20% after 2.5 s.

**The relationship between the TENG’s active area and wind force levels:** Optical communication is an efficient and low-energy communication method. Replacing Bluetooth communication with optical communication obviously helps to complete related low-energy transmission tasks. This communication technology has a high degree of maturity. For example, with the continuous development of visual processing, optical nodes such as surveillance cameras can already be effectively utilized. Therefore, the development of self-powered optical communication technology does not require complex electronic device design; it only relies on LED lights with a common CCD to complete the relevant communication process. Another advantage of this open optical communication system is that its signal is not only be received by the preset electronic system. In addition, the human eye can quickly process and analyze its signal, which virtually improves the application freedom of the related fully self-powered system.

**Figure 6:**
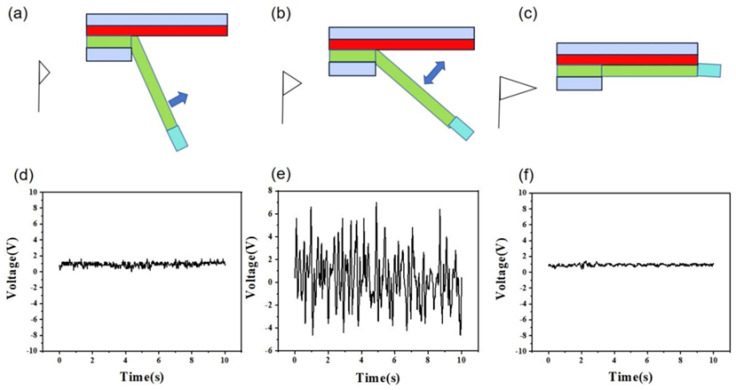
Schematic diagrams of the operating modes (**a**–**c**) for the flag-shaped triboelectric nanogenerator (TENG) for wind speed measurement and the corresponding open-circuit voltages (**d**–**f**).

In the process of investigating the contact-separation mechanism of wind-driven PVDF and PA6 fiber membranes, we observed the effect of wind force on the performance of the triboelectric nanogenerator (TENG). Through a series of experiments, we analyzed the relationship between wind force and TENG output in detail and revealed the underlying physical mechanisms. First, when the wind force is zero or very small, the oscillation amplitude of the PVDF fiber membrane is extremely limited. Since the wind force is insufficient to overcome the gravity of the counterweight, the membrane cannot stably contact and separate from the PA6 fiber membrane. As shown in Figure 6a, the TENG output is nearly zero at this stage due to the lack of sufficient energy to drive the contact-separation process. As the wind force increases, the situation begins to change. When the wind force reaches a certain threshold, the vertical component of the wind force on the fiber membrane balances the vertical component of the counterweight’s gravity. This equilibrium allows the PVDF fiber membrane to oscillate with just enough amplitude to undergo unstable contact with the PA6 membrane, enabling continuous contact-separation events. As depicted in Figure 6b, this process provides a stable energy source for the TENG, generating consistent electrical signals (output shown in Figure 6e). However, when the wind force exceeds a critical limit, the vertical component of the wind force on the fiber membrane surpasses the vertical gravitational component of the counterweight. This causes the PVDF membrane to lose stable contact-separation with the PA6 membrane, and they may even fail to separate after contact. As illustrated in Figure 6c, this negatively impacts TENG performance, causing the output to gradually decrease and approach zero (Figure 6f).

Here, the wind speed threshold is correlated with the size and mass of the TENG. According to fluid dynamics principles such as Bernoulli’s equation, wind pressure and wind speed follow the equation:

$$P_{wind}=\frac{1}{2}\rho v_{wind}^{2}$$

When the wind speed reaches the operational threshold, the aerodynamic force acting on the triboelectric nanogenerator (TENG) balances its gravitational force, yielding the critical wind speed, which is noted as follows:

$$v_{0}=\sqrt{\frac{2mg}{\rho S}}$$

Here, *S* represents the contact-separation area of the TENG. In this experiment, the flag-type TENG exhibited a contact-separation area of **56 cm^2^** and a mass of **2.798 g**, with an experimentally determined wind speed threshold of approximately **2.7 m/s**. 

**Figure 7:**
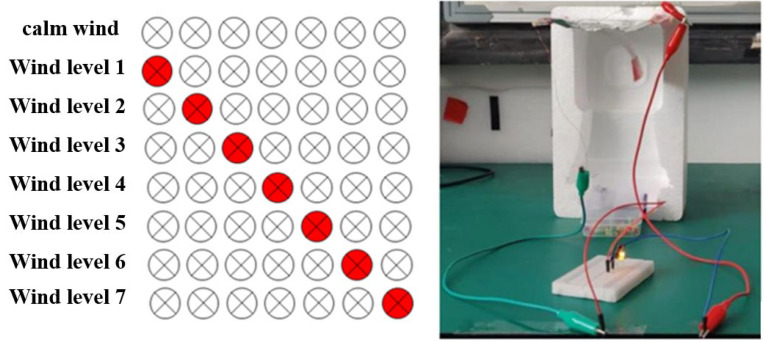
The triboelectric nanogenerator (TENG) for wind speed measurement lights up LED bulbs.

Analysis of experimental data reveals a direct correlation between the wind speed threshold for continuous contact separation and the counterweight’s gravitational force. The continuous contact-separation operation of the TENG arises from the resistance between wind pressure and the torque of the PVDF fiber membrane. The fixed torque allows the TENG to enter this operational mode only when the wind pressure reaches a specific value. As shown in Figure 7, we integrated the TENG with an LED bulb to form a self-powered sensor. When the flag-shaped wind-driven TENG operates in continuous contact-separation mode, it lights up the LED bulb. This realizes the conversion from wind energy to electrical signals and then to optical signals, enabling wind-pressure indication using LEDs.

**Figure 8:**
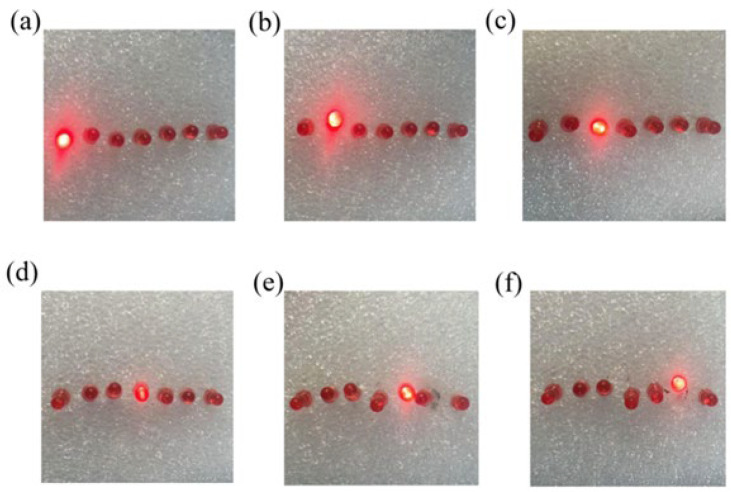
(**a**–**f**) Illumination states of LED bulbs lit by the self-powered sensor array under different wind forces.

While keeping the mass of the counterweight and the width of the PVDF membrane constant, the torque of the PVDF membrane increases with its length. A larger torque corresponds to a lower wind pressure required for the TENG to enter the continuous contact-separation operating mode. By adjusting the length of the PVDF membrane, we can set the wind pressure threshold at which the self-powered sensor’s LED bulb lights up. By assembling self-powered sensors with PVDF membranes of different lengths into a sensor array, we achieve wind speed measurement through wind-pressure LED indication. As shown in Figure 8, each LED bulb is connected to a TENG with a PVDF membrane of decreasing length from left to right. As the membrane length shortens, the torque decreases, and the corresponding wind pressure threshold increases. When the wind speed gradually rises, the lit LED bulbs move from left to right accordingly.

By tuning the torque of the PVDF membrane, we developed a self-powered sensor array based on a flag-shaped triboelectric nanogenerator with high fiber order, realizing a self-powered sensing system for wind-pressure LED indication. This design simplifies the circuit, eliminates the need for traditional control chips, reduces power consumption, and enhances system reliability. Additionally, the system can detect the wind force generated by passing trains, providing critical support for protecting the structure of railway canopies.

## 4. Conclusions

In this work, a flag-type TENG was prepared using in situ electrospinning technology combined with the silicone oil paper isolation method. The TENG can collect wind fluctuations and light up LEDs for optical communication. By controlling the area of the flag TENG, the self-powered system for different wind responses is designed, and the self-powered sensing capability for different winds is generated by combining the systems. The flag-type TENG was found to have an output power of 11.14 mW/m^2^. The effective binding of the flag connecting side can be attributed to the good van der Waals interaction generated by in situ preparation. The several grids method test verified the good mechanical bonding generated by in situ preparation. The system has been proven to work independently of the battery, wiring, or a second TENG and is therefore a fully self-powered system. Compared with existing flag-structured triboelectric nanogenerator (TENG) devices, the in situ electrospun flag-type TENG developed in this study demonstrates enhanced interfacial bonding strength and superior charge transfer efficiency, significantly improving its output performance. These advantages stem from the homogeneous fiber-substrate integration achieved through the in situ electrospinning process, which optimizes both mechanical interlocking and charge-trapping capabilities at the interface. This work lays a foundation for the future design of the Internet of Things based on self-powered systems.

## Data Availability

The original contributions presented in this study are included in the article/Supplementary Material. Further inquiries can be directed to the corresponding author(s).

## Supplementary Materials

The following supporting information can be downloaded at: https://www.mdpi.com/article/10.3390/nano15110839/s1.
